# Effect of long-term proton pump inhibitors on phosphocalcium metabolism and bone mineral density

**DOI:** 10.2144/fsoa-2023-0198

**Published:** 2024-05-24

**Authors:** Hend Smaoui, Lassaad Chtourou, Dana Jallouli, Samar Ben Jemaa, Iheb Karaa, Mouna Boudabbous, Manel Moalla, Hela Gdoura, Leila Mnif, Ali Amouri, Rim Akrout, Fatma Ayadi, Sofien Baklouti, Nabil Tahri

**Affiliations:** 1 Department of Gastroenterology & Hepatology, Hedi Chaker University Hospital, Sfax, Tunisia; 2 Laboratory of Biochemistry, Habib Bourguiba University Hospital, Sfax, Tunisia; 3 Department of Rhumatology, Hedi Chaker University Hospital, Sfax, Tunisia

**Keywords:** bone mineral density, calcemia, magnesemia, osteopenia, proton pomp inhibitors

## Abstract

**Aim:** Although Proton pump inhibitors (PPIs) are well-tolerated, their long-term use may be associated with decreased bone mass. **Methods:** This is a case-control study including patients treated with PPIs (>1 year) and control subjects who have not received PPIs treatment. **Results:** A total of 90 patients and 90 matched controls were included. PPIs use was associated with hypocalcemia and hypomagnesemia. Vitamin D3 deficiency and hyperparathyroidism were associated with PPIs use. Long-term PPIs use was significantly associated with decreased bone density. Risk factors of decreased bone mineral density (BMD) included age >50 years, menopause, lack of sun exposure, double PPIs dose, daily intake, post-meal intake and association with a mucoprotective agent. **Conclusion:** Our results highlight the risk of decreased BMD in patients on long-term PPIs treatment.

## Plain language summary

In this study, we investigated the impact of long-term use of proton pump inhibitors (PPIs) on bone health. Examining 90 patients on PPIs for over a year and matching them with control subjects, we found that PPIs were linked to lower bone density. Factors such as age over 50. menopause, limited sun exposure, higher PPIs dosage, daily intake, post-meal usage and combining PPIs with a mucoprotective agent were associated with decreased bone mineral density. Our findings emphasize the concern of reduced bone density in individuals undergoing prolonged PPIs treatment.

## Abstract (plain language summary)

Association with bone health: the clinical trial underscores the well-established association between long-term proton pump inhibitor (PPI) use and adverse effects on bone health, including decreased bone mineral density (BMD).

Risk factors for decreased BMD: the study identifies specific risk factors contributing to reduced BMD in patients on long-term PPI treatment. Notable factors include age over 50 years, menopausal status, lack of sun exposure, double PPI dose, daily intake, post-meal administration, and the concurrent use of mucoprotective agents.

Magnitude of the study: the research encompasses a substantial sample size, involving 90 patients on long-term PPI treatment and 90 matched controls. This robust dataset allows for a comprehensive exploration of various factors influencing bone health.

Biochemical disturbances: the trial meticulously examines phosphocalcic assessment, parathyroid hormone (PTH) levels, 25-hydroxy vitamin D levels, and osteodensitometric data. This detailed biochemical analysis enhances the understanding of the physiological changes associated with PPI use.

Hypomagnesemia and hypocalcemia: the study highlights a significant association between PPI use and hypomagnesemia, contributing to the understanding of the impact of PPIs on magnesium levels.

Age and menopause Impact: the findings underscore the significance of age, particularly those over 50 years, as a critical factor in decreased BMD associated with PPI use. Menopausal status among women is identified as another key determinant of bone health in this context.

Specific PPI conditions: the trial reveals specific conditions under which the risk of bone demineralization is elevated with PPI use. These conditions include regular use, double dose, daily intake, post-meal administration, and combination with a mucoprotective agent.

Multivariate analysis: through multivariate analysis, the study pinpoints that a decrease in BMD is significantly more observed in patients aged over 50 years, even after adjusting for various factors such as age, physical activity, menopause, and PPI treatment modalities.

Caution in prescription: the research calls for a more cautious approach in prescribing PPIs, especially in long-term use and in patients over the age of 50. given the identified risks to bone health. The study suggests that healthcare providers should carefully weigh the potential benefits against the risks associated with prolonged PPI use.

No increase in fracture risk: contrary to some previous studies, this trial does not find an increased risk of vertebral or femoral neck fractures associated with PPI use, emphasizing the complexity of the relationship between PPIs and bone health.

Proton pump inhibitors (PPIs) are potent gastric antisecretory agents. In clinical practice, PPIs have been one of the most prescribed treatment since their commercialization in 1989 [1]. There are several subtypes of PPIs [2]. Although they work in the same way, there are slight differences among them in terms of their pharmacokinetic properties, metabolism and clinical indications approved by the US FDA.

Therefore, PPIs are commonly used to treat various gastrointestinal disorders, such as gastric and duodenal ulcers, gastroesophageal reflux disease (GERD) and Zollinger-Ellison syndrome. Additionally, they also serve as prophylactic agents in non-steroidal anti-inflammatory drug (NSAID) users to prevent gastric ulcers and hemorrhagic complications [3]. When combined with antibiotics, PPIs are also an integral part of the eradication treatment for *Helicobacter pylori* (*H. pylori*) [4]. Moreover, PPIs are used for functional dyspepsia and long-term management of Barrett's esophagus [5–7].

However, long-term use of PPIs can be associated with certain adverse effects. Abnormalities in phosphocalcic metabolism, including bone demineralization and decreased bone mineral density (BMD), as well as an increased risk of fractures [8]. This is a public health issue that affects quality of life and influences short-term and long-term morbidity and mortality in the population [9].

## Methods

This is a case-control, descriptive, and analytical study conducted at our department of Gastroenterology. The study took place over a period of 13 months from January 2021 to January 2022.

We recruited patients divided into two groups:

- Patient group: including adult patients with a chronic condition treated with a PPIs for more than one year. Omeprazole and Esomeprazole were the only PPIs available for our patients;
- Control group: including healthy controls who do not take PPIs, matched to the patient group based on age and gender.

We excluded subjects from both groups who had: a history of gastric surgery, a personal or family history of osteoporosis, a reported calcium or vitamin D supplementation and an underlying secondary causes of osteoporosis (including drugs that may interfere with calcium metabolism).

We collected epidemiological data, indication for PPIs treatment, methods of PPIs administration, clinical symptoms, phosphocalcic assessment, parathyroid hormone (PTH) measurement, 25-hydroxy vitamin D level, and osteodensitometric data.

The data were analyzed using SPSS software version 22.0. For qualitative variables, we calculated simple frequencies and relative frequencies (percentages). Quantitative variables were described using means and standard deviations (SD), or medians and ranges (minimum and maximum). In a univariate analysis, we compared the parameters of the phosphocalcic assessment and the osteodensitometric profile between the two groups. Then, we examined factors (epidemiological, clinical, histological and biological) correlated with the biological and densitometric disturbances in each group. The comparison of two means was performed using the Student's *t*-test if the application conditions were met. The comparison of percentages was carried out using Pearson's chi-square test. The Fisher's exact test was used if any of the calculated theoretical frequencies were less than five. Furthermore, we conducted a multivariate analysis using regression analysis. For all tests conducted, differences were considered statistically significant at a p-value <0.05.

## Results

The average age of our patients was 55 ± 12.22 years, ranging from 18 to 80 years. Our population consisted of 40 males (44.4%) and 50 females (55.6%). The sex ratio was 0.8. Seventeen patients (18.9%) were smokers, with an average consumption of 21.53 ± 12.32 pack-years (PY). Nine patients (10%) had hypertension, two had asthma and two had anemia. Among the 50 women in the study population, 25 were menopausal. The average age of menopause was 49.5 ± 2.48 years. Among the 50 women in the study population, 42 wore veils (84%). The median sun index was 1.96, ranging from 0.09 to 17.64. Sixty-six patients were sedentary (74%). Among the active individuals, 24 engaged in moderate physical activity (26%), and no one had high-level activity. The average body mass index (BMI) was 28.54 ± 4.01 kg/m^2^.

We investigated several indications prevalent among patients in the given population. Gastroesophageal reflux disease (GERD) emerged as the most common condition, with 48 patients accounting for 53.3% of the study. Additionally, we found 17 cases (18.9%) of GERD complicated by peptic esophagitis. Dyspeptic symptoms were observed in 23 patients, representing 25.6% of the population. Furthermore, NSAID use was reported in only two patients, comprising 2.2% of the sample.

The endoscopic lesions were as follows: congestive and/or ulcerative gastropathy in 31.1% of patients, peptic esophagitis in 16.66% of cases, bulbar ulcer in 13.3% of cases, hiatal hernia found in 5.6% of patients, coexistence of bulbar ulcer and peptic esophagitis in 2.22% of patients. *H. pylori*-associated gastritis was described in 48,9% of patients.

Table 1 summarizes the characteristics of PPIs usage in the study population.

**Table 1.** Characteristics of PPI usage in the study population.

Proton pump inhibitor		Percentage (%)
Therapeutic class	Oméprazole	81.1%
	Esoméprazole	18.9%
Dose	Single	93.3%
	Double	6.7%
Frequency of administration	Daily	78.9%
	Every 2 days	7.8%
	Every 3 days	8.9%
	Every 7 days	4.4%
Consistency of administration	Regular	64.4%
	Irregular	35.6%
Timing of administration	Before meals	44.4%
	After meals	55.6%
Combination with antacid and/or mucoprotective agent	Yes	87.8%
	No	12.2%

The average calcium level was 2.33 ± 0.16 mmol/l. Out of the total number of patients, 57 individuals had normal calcium levels, which accounts for 63.33% of the study population. The average phosphorus level was 1.08 ± 0.18 mmol/l. The 84 individuals had normal phosphorus levels, which accounts for 93.33% of the study population. The average magnesium level was 0.82 ± 0.09 mmol/l. Hypomagnesemia was observed in only 10% of the patients. The average vitamin D level was 19.72 ± 10.79 mmol/l. Eighty patients had a deficiency in vitamin D3. The average PTH level was 66.48 ± 32.4 mmol/l. The majority of patients had a normal PTH level, accounting for 55.6% of the study population.

Out of the total number of patients, 38 individuals had a normal bone mineral density. Osteopenia was described in 46 patients and osteoporosis was found in six individuals.

Osteopenia/osteoporosis was significantly more common in the patient group (p = 0.0001). Lower calcium levels were significantly associated with the use of PPIs (p = 0.048). The same was observed for magnesium levels (p = 0.029). Additionally, PTH levels were significantly higher in patients taking long-term PPIs (p = 0.005), as well as vitamin D3 levels (p = 0.0001).

Hypomagnesemia was significantly associated with hypocalcemia (p = 0.0001). Calcium levels were not influenced by other epidemiological and clinical factors. There was no statistically significant relationship between these parameters and phosphorus or magnesium levels.

Vitamin D3 deficiency was not correlated with any epidemiological, clinical, or biological factors.

Hyperparathyroidism was observed in patients over 50 years old (p = 0.04) and in the case of hypocalcemia (p = 0.034). A low osteodensitometric profile (osteopenia or osteoporosis) was significantly associated with age >50 years (p = 0.036), menopause (p = 0.021), and wearing a veil (p = 0.005). There was a significant increase in the risk of bone demineralization with PPIs use under the following conditions: regular use (p = 0.014), double dose (p = 0.037), daily use (p = 0.002), administration after meals (p = 0.028), and combination with a mucoprotective agent (p = 0.007). The specific class of PPIs, duration of use, calcium levels, phosphorus levels, magnesium levels, vitamin D levels and PTH levels did not affect the results of the osteodensitometry.

Table 2 shows the comparison of clinical, biological and osteodensitometric data in the study population.

**Table 2.** Comparison of clinical, biological, and osteodensitometric data in the study population.

Parameter		Patients (N = 90)	Controls (N = 90)	p-value
Epidemiological	Age mean (years)	54.77 ± 12.21	55.17 ± 12.4	0.83
	Gender (M/F)	40/50	37/53	0.56
	Tobacco (yes/no)	73/17	78/12	0.31
	Alcohol (yes/no)	87/3	87/3	1
	Menopause (no/yes)	25/25	21/32	0.29
	Veil-wearing (no/yes)	8/43	12/41	0.39
Clinical	Mean BMI (kg/m^2^)	28.54 ± 4.01	28.05 ± 3.95	0.47
	Mean corrected calcium (mmol/l)	2.24 ± 0.16	2.27 ± 0.18	0.10
	Calcium (normal/high/low)	58/32/0	70/20/0	0.048^†^
	Mean PH (mmol/l)	1.08 ± 0.18	1.00 ± 0.07	0.002^†^
	Phosphoremia (normal/low)	84/6	80/10	0.29
	Mean magnesium (mmol/l)	0.82 ± 0.09	0.93 ± 0.12	0.0001^†^
	Magnesium (normal/high/low)	81/9/0	88/2/0	0.029^†^
	Mean PAL (UI/L)	79.56 ± 22.07	77.65 ± 23.1	0.65
	Mean vitamin D3 (ng/ml)	19.72 ± 10.79	14.77 ± 10.52	0.001^†^
	Vitamin D3 (normal/low)	10/80	7/83	0.44
	Mean PTH (pg/ml)	66.48 ± 32.4	55.08 ± 31.8	0.005^†^
	PTH (normal/ high)	50/40	67/23	0.008^†^
	Mean hemoglobin (g/dl)	13.14 ± 1.15	12.69 ± 1.3	0.03^†^
	Ferritinemia (μg/l)	68.02 ± 70.01	65.31 ± 85.3	0.79
	TSH (mU/l)	2.32 ± 0.93	1.92 ± 1.04	0.23
Osteodensitometry	Bone Mass (normal/osteopenia/osteoporosis)	38/46/6	59/23/1	0.0001^†^
	Bone Mass (normal/low)	38/52	59/24	0.0001^†^
	Mean Spine T-score (DS)	-0.61 ± 1.53	0.05 ± 1.13	0.003^†^
	Mean Spine Z-score (DS)	-0.35 ± 1.37	0.13 ± 1.5	0.03^†^
	Mean Femur T-score (DS)	-0.55 ± 0.17	-0.26 ± 0.25	0.09
	Mean Femur Z-score (DS)	0.05 ± 1.04	0.32 ± 1.17	0.11

† p-values that were considered statistically significant.

Table 3 shows the various risk factors for osteopenia in the population treated with PPIs in univariate analysis

**Table 3.** Risk factors for osteopenia in the population treated with PPIs in univariate analysis.

DMO		Low	Normal	p-value
Epidémiological	Age (<50ans/ >50ans)	9/43	14/24	0.036^†^
	Gender (M/F)	22/30	18/20	0.63
	Average solar index (h/week)	2.12	1.75	0.65
	Physical activity (sedentary/average)	42/10	24/14	0.062
	Menopause (no/yes)	11/19	14/6	0.021^†^
	Veil wearing (no/yes)	1/29	7/13	0.005^†^
Clinical	BMI (kg/m^2^)	28,84	28,13	0.41
	PPIs class (omeprazole/esomeprazole)	45/7	28/10	0.12
	Regularity (regular/irregular)	39/19	19/19	0.014^†^
	Daily dose (simple dose/double dose)	46/6	38/0	0.037^†^
	Frequency (every day/ ≥1 day/2)	47/5	24/14	0.002^†^
	Duration (mean)	94.85	99.13	0.74
	Schedule (before meal/ after meal)	18/34	22/16	0.028^†^
	Combination with an antacid (no/yes)	47/5	32/6	0.51
	Combination with a mucoprotective agent (no/yes)	50/2	29/9	0.007^†^
Histological	Presence of HP (no/ yes)	2/26	1/18	0.67
	Calcuim (normale ou élevée/basse)	31/21	27/11	0.26
	Phosphoremia (normal/low)	51/1	33/5	0.08
	Magnesemia (normal/low)	46/6	35/3	0.73
	VitD3 (mean)	18.15	21.88	0.14
	Vit D3 (normal/low)	3/49	7/31	0.083
	PTH (mean)	65,41	67,93	0.71
	PTH (low or normal/high)	29/23	21/14	0.96

† p-values that were considered statistically significant.

In multivariate analysis, a decrease in BMD was significantly more observed in patients aged over 50 years (p = 0.032), with an odds ratio of 2.78. This was after adjusting for age, physical activity, menopause, wearing a veil, PPIs class, treatment modalities (regularity, daily dose, frequency, timing of administration, combination with a mucoprotective agent) and vitamin D levels (Table 4).

**Table 4.** Risk factors for a decrease in bone mineral density in multivariate study.

Risk factor	p-value	OR
Age >50ans	0.032^†^	2.78
Physical activity	0.114	–
Menopause	0.280	–
Veil wearing	0.128	–
PPIs class	0.114	–
Regularity	0.051	–
Daily dose	0.999	–
Frequency	0.404	–
schedule	0.754	–
Combination with a mucoprotective agent	0.056	–
Vitamine D3	0.838	–

† p-values that were considered statistically significant.

## Discussion

Our results highlight the risk of decreased bone density in patients on long-term PPIs treatment without an increase in fracture risk.

In most studies analyzing bone modifications in patients on long-term PPIs therapy, as well as in our work, magnesium and calcium levels were found to be low.

A large Italian study conducted in 2020 by Recart DA *et al.*, which included 236 patients on PPIs therapy for more than 6 months, showed that patients on long-term PPIs use had a high prevalence of hypomagnesemia [10].

Calcium levels were lower in the group of patients suffering from hypomagnesemia, as magnesium deficiency induces a state of resistance to PTH. Phosphorus levels were normal but lower in subjects with hypomagnesemia.

Similarly, in a recent literature review published in 2019 by P. Cabras *et al.*, which included 58 patients and 40 studies, hypocalcemia and hypomagnesemia were observed in patients who were consulting for neurological, muscular and/or cardiac rhythm disorders and had been on PPIs therapy for more than a year without any other identifiable causes explaining the clinical symptoms [11].

Luk CP *et al.* published a literature review based on 66,102 cases reported to the Food and Drug Administration (FDA). All PPIs were found to be a risk factor for hypomagnesemia. Pantoprazole and Omeprazole were associated with a higher risk of hypomagnesemia compared with Esomeprazole and Rabeprazole. Hypocalcemia frequently coexisted with hypomagnesemia [12]. Similarly, in a literature review published in 2020. Wakeman M. concluded that long-term use of PPIs was associated with hypomagnesemia and hypocalcemia in some cases [13]. Additionally, a study conducted in 2015 by Janett S. *et al.* examined 60 patients treated with PPIs for more than a year, presenting severe hypomagnesemia associated with hypocalcemia and hypokalemia, along with neuromuscular and cardiac disorders. The link between PPIs treatment and hypomagnesemia/hypocalcemia was strongly established, with subjects over 50 years old being the most vulnerable to these metabolic disorders [14].

However, a case-control study published in 2013 by Koulouridis I. *et al.*, did not show a significant hypomagnesemia in the patients. The risk factors identified were diabetes and concomitant diuretic therapy, and there were no significant differences between different classes of PPIs [15].

The literature data about phosphate levels are controversial. Each of the previous studies reported results within the normal range [10–12,14]. On the other hand, on a recent study by Losurdo *et al.* phosphate levels were significantly lower in PPIs users versus controls. That's probably related to the effect of PPIs, who can inhibit osteoclast proton pumps, reducing bone calcium phosphate reabsorption [16].

Our study's findings were consistent with those reported in the literature. Indeed, the average magnesium levels were significantly lower in patients taking PPIs for an extended period, and the number of patients with hypomagnesemia was significantly higher in this group. The number of patients with hypocalcemia was significantly higher in the patient population compared with the controls. This effect was similar for all PPIs, with no significant differences suggesting a class effect. The treatment's modalities did not influence the results, and no risk factors were identified for hypocalcemia or hypomagnesemia. However, we did not observe a decrease in phosphorus levels in our patients compared with the control population.

According to the pathophysiological mechanism proposed by Yang YX *et al.*, hypocalcemia secondary to PPIs use can lead to secondary hyperparathyroidism [17]. Another mechanism of secondary hyperparathyroidism is hypergastrinemia resulting from a decrease in gastric pH. This was validated by a study in 2015 by Hinson AM *et al.*, which included 40 subjects on long-term PPIs therapy and 40 control subjects. The measurement of Parathyroid Hormone (PTH) levels was significantly higher in patients exposed to PPIs compared with the control group [18]. Secondary hyperparathyroidism can cause hyperplasia of the parathyroid glands, leading to autonomous and uncontrolled secretion of PTH.

Similarly, in our study, we demonstrated a significant elevation of PTH levels in patients compared with healthy subjects. Only two risk factors were associated with this elevation: age over 40 years and hypocalcemia. The class of PPIs and the dosing regimen did not influence the PTH levels.

On the contrary, Fatuzzo P *et al.* reported a case in 2017, involving a patient who had been treated with PPIs for 20 years and was hospitalized for severe hypocalcemia and hypomagnesemia related to the treatment [19]. The PTH levels were found to be very low. The conclusion was that hypomagnesemia is a reversible cause of hypoparathyroidism [20,21]. It directly leads to hypocalcemia, and detecting hypomagnesemia is crucial in this situation as it determines the prognosis and management of hypocalcemia.

Another literature review conducted in 2013 by Famularo G *et al.*, which included 32 patients exposed to PPIs for prolonged periods, mostly exceeding 1 year and up to over 5 years, showed that all patients presented the following profile: hypomagnesemia, hypocalcemia, low to normal PTH levels, and normal vitamin D levels, suggesting the role of hypomagnesemia in hypoparathyroidism [22]. Hypomagnesemia was refractory to oral or parenteral supplementation but rapidly normalized after stopping PPIs, with relapse occurring after re-exposure to PPIs.

PPIs are suggested as a probable etiology of decreased vitamin D due to hypomagnesemic hypoparathyroidism reported in some sources of the literature [23].

For instance, an article published in 2015 by Attwood SE. *et al.* reported the results of a randomized controlled study (LOTUS) conducted between 2001 and 2009 in 11 countries (Belgium, Denmark, France, Germany, Austria, Iceland, Italy, Norway, Sweden, the UK and the Netherlands) comparing patients treated for GERD with two therapeutic approaches: Esomeprazole (266 patients, treatment lasted an average of 5 years) and surgical intervention (248 patients). The study aimed to determine the adverse effects of PPIs (Esomeprazole). There was no difference in calcium and vitamin D levels between patients on PPIs and patients who underwent surgery [24]. Similarly, a literature review in 2014 by Anne Claire B [25] which included four publications studying the effects of PPIs on vitamin D did not show any signs of vitamin D deficiency [26–29].

Our results align with these findings. The use of PPIs was not identified as a risk factor for decreased vitamin D levels in our patients.

The article published in 2017 by Vaezi MF. proposed that the mechanism of bone loss with subsequent risk of fracture during PPIs use involves a reduction in gastric acidity with hypergastrinemia [30]. The reduction in gastric acidity can lead to malabsorption of calcium and vitamin B12, subsequently causing secondary hyperparathyroidism. Chen CH. *et al.* confirmed this hypothesis in a large cohort published in 2016 [31]. The risk of osteoporosis was significantly higher in patients compared with controls (p < 0.001). The risk factors associated with osteoporosis were age, female gender, GERD, hypertension, heart failure, hyperlipidemia, cirrhosis, corticosteroid use and anticonvulsants. This finding was also confirmed in an Australian cohort comparing 2328 women on long-term PPIs therapy with 2104 age-matched women [32]. A statistically significant difference was observed between the two groups, further supporting this hypothesis. The risk was higher for esomeprazole and rabeprozole compared with other PPIs. Overweight/obesity, general health impairment and corticosteroid use were correlated with bone damage. These findings have also been reported in a literature review published in 2012 by Lau YT. *et al.* [3], which included 14 studies comparing the risk of reduced BMD or fractures between PPIs users and non-users, or between current and past PPIs users, including eight case-control studies and six cohort studies [33–46]. The risk of fracture was evaluated at the hip in 11 studies, the spine in five studies, and the wrist in three studies. The effect of PPIs use on BMD was evaluated in three studies [33,40,43]. Eight studies found an increased risk of hip fracture [34,36,39,41,42,44–46]. Five studies found an increased risk of spine fracture [34,35,37,40,42]. One study found an increased risk of wrist fracture [40]. However, none of the three studies on BMD found any association with PPIs use. A dose-response relationship measured by duration or dosage was demonstrated in four studies [36,41,45,46]. These four studies also showed that increasing daily dosage increased the risk of fracture. The update of the American Gastroenterological association (AGA) guidelines on PPIs use is based on similar research [1]. A small cross-sectional study reported that PPIs were associated with lower trabecular BMD but not cortical BMD [47]. On the other hand, a cohort study reported a decrease in vertebral T-score without any effect on the femoral T-score [45]. In the latter study, only female gender, overweight and sedentary lifestyle were independent risk factors for osteopenia. Currently, there is insufficient data to systematically monitor BMD in PPIs users. A recent literature review published in 2022 by Les pessailles E *et al.* reported various findings from several studies investigating the relationship between PPIs and bone profile [48]. The reduction in BMD associated with PPIs use has been studied in well-known cohort studies such as the Study of Osteoporotic Fractures (SOF), the Osteoporotic Fractures in Men (MrOs) study, and the Women's Health Initiative (WHI) studies [49–51]. None of these studies found changes in BMD affected by PPIs exposure. These findings are consistent with the conclusions of the SWAN study, which showed no association between PPIs use and BMD loss [52]. This is confirmed in several recent systematic reviews [52–54]. Although based on a limited number of prospective or retrospective cohorts, the conclusion was consistently the same: there was no significant change in BMD between PPIs users and non-users. However, Aleraij *et al.* demonstrated in their meta-analysis that there was a statistically significant reduction in mean BMD among PPIs users [55,56].

A literature review published in 2019 by Thong BS *et al.*, including 18 studies, concluded that the use of proton pump inhibitors (PPIs) was a risk factor for pathological fractures in 14 publications [57–70]. However, an association between PPIs use and fractures was excluded in four other studies [71–74].

A meta-analysis conducted by Poly *et al.*, including 24 studies (nine cohorts and nine case-control studies), showed an increased risk of femoral neck fracture in patients receiving PPIs with a relative risk (RR) of 1.2 [75]. However, intermediate and long-term use had a nearly similar and higher risk of hip fracture. Low, medium, or high doses of PPIs were associated with 17%, 29% and 30% increased risk of upper femoral fractures, respectively. An increased risk of upper femoral fracture was observed with Rabéprazole, Pantoprazole, and Omeprazole, but no association was observed with Esomeprazole and Lansoprazole.

Sugiyama T. *et al.* suggest that the risk of bone fracture in subjects taking PPIs is minimal, given that bone has a great capacity to adapt to metabolic changes. However, they highlight the frequency of falls in the elderly, often prescribed PPIs, which can be an added cause of morbidity [76].

A literature review published in 2014 concluded that PPIs prescription is a risk factor for pathological bone fractures. This association remains modest, as studies are more likely to be due to confusion rather than a causal effect [77]. A meta-analysis conducted in 2011 by Ngamruengphong S *et al.* showed a modest association between PPIs use and an increased risk of upper femoral and vertebral fractures, but no evidence of a duration effect in subgroup analysis [78].

Contrary to all these studies, our study did not show an increased risk of vertebral fractures or femoral neck fractures, even in subjects with osteopenia or osteoporosis.

The innovative aspect of this clinical trial lies in its meticulous examination of the association between proton pump inhibitors (PPIs) and bone homeostasis, particularly focusing on an extensive range of factors contributing to decreased bone mineral density (BMD). While previous clinical trials have established a connection between PPI use and bone-related issues, this study advances the field by providing a comprehensive investigation into the various parameters influencing bone health.

## Conclusion

Our results highlight the risk of decreased bone density in patients on long-term PPIs treatment without an increase in fracture risk. This calls for a more cautious prescription of these medications, especially in long-term use in patients over the age of 50.
